# Cardioncological Approach for Trastuzumab Therapy in Breast Cancer Patients With Cardiotoxicity: Impact on Adherence and Clinical Outcome

**DOI:** 10.3389/fphar.2020.01190

**Published:** 2020-08-04

**Authors:** Iacopo Fabiani, Carlo Maria Cipolla, Nicola Colombo, Daniela Cardinale

**Affiliations:** ^1^ Cardioncology Unit, European Institute of Oncology, I.R.C.C.S., Milan, Italy; ^2^ Cardiology Division, European Institute of Oncology, I.R.C.C.S., Milan, Italy

**Keywords:** trastuzumab, breast cancer, heart failure, ejection fraction, cardiotoxicity, systolic dysfunction

## Abstract

**Background:**

Treatment with Trastuzumab is associated with cardiotoxicity. If Trastuzumab could be administered in a safe manner to patients who develop a reduced left ventricular ejection fraction (EF) of < 50% remains poorly understood.

**Objective:**

To evaluate the impact of a cardioncological approach in terms of adherence and continuation of oncological therapy with Trastuzumab.

**Methods and Results:**

Internal databases of candidates for trastuzumab chemotherapy with evidence of cardiotoxicity according to echocardiographic criteria were retrospectively evaluated. Eighty-four female patients (age 51.7 years, 95% CI 49.5–53.8), were finally included. Patients were divided to receive a standard (n 27) or cardioncological (n 57) scheme. Baseline EF values were within normal limits (60.9, 95% CI 60 - 61.9%; p=0.5 between groups). The nadir of EF observed during trastuzumab therapy was more pronounced in the standard care group (40.6, 95% CI 37.3–43.9% vs. 46.3, 95% CI 44.3–48.3%; p=0.002). At re-challenge, after cardiotoxicity detection, all patients in the cardioncological arm resumed and completed trastuzumab therapy (p<0.0001). An overall reduction of EF was observed at the final evaluation (p <0.0001 vs. baseline). Cardioncological approach was the only independent determinant of ΔEF from baseline to final evaluation (R^2^0.12; p=0.004). We observed a total of 13 (15%) HF events, seven (26%) in the standard, and six (10%) in the cardioncological approach group (p =0.1). Patients in the cardioncological approach arm had a better outcome (Log Rank Chi-squared 4.89; p=0.02).

**Conclusions:**

A targeted cardioncological approach, in patients with evidence of cardiotoxicity during HER-2 inhibitor therapy, could favorably influence the oncological management of breast cancer patients, reducing the adverse cardiovascular impact of chemotherapy.

## Introduction

More than 20% of breast cancers express human epidermal growth factor receptor-2 (HER-2) (Slamon et al., 1987; Slamon et al., 1989).

Trastuzumab has already shown to improve patients’ survival (Piccart-Gebhart et al., 2005; Romond et al., 2005; Slamon et al., 2011). However, trastuzumab-containing regimens are associated with cardiotoxicity, in particular after anthracyclines (ACs) administration, with the development of reduced left ventricular ejection fraction (EF) (Slamon et al., 2011; Romond et al., 2012; Tarantini et al., 2012; Goldhirsch et al., 2013).

In this respect, full recovery of left ventricular systolic function is not always achieved, despite interruption of the drug (Guarneri et al., 2006).

Currently, guidelines for cardiac surveillance recommend an EF evaluation before and during trastuzumab therapy (Mackey et al., 2008; Dang et al., 2016; Armenian et al., 2017), in particular in cases of early cardiotoxicity (Yu et al., 2015a; Seferina et al., 2016).

Recent trials confirmed that the HF rate is usually limited to the initial treatment period (Romond et al., 2012; Advani et al., 2016; Cameron et al., 2017), and retrospective data suggest that the continuation of this drug may be safe in asymptomatic with EF reduction (Yu et al., 2015b).

In this retrospective study, we evaluated the impact of a dedicated cardioncological approach in terms of adherence and continuation of oncological therapy with Trastuzumab.

## Methods

Internal institutional echocardiographic databases (Jan 2010–Jan 2019) of female patients with breast cancer, candidates for trastuzumab chemotherapy, with evidence of cardiotoxicity according to previously defined echocardiographic criteria (absolute decrease of at least 10% from baseline EF to less than 55% during the trastuzumab treatment period without symptoms of HF), were retrospectively evaluated. In all patients, trastuzumab therapy had been (temporarily—subsequent rechallenge—or permanently) interrupted after developing cardiotoxicity, while waiting for cardiological evaluation.

The study was carried out in accordance with the Declaration of Helsinki. The local Ethics Committee approved the study (Comitato Etico degli I.R.C.C.S. Istituto Europeo di Oncologia e Centro Cardiologico Monzino—Milano—Italy: N. R709/18-IEO 750).

Indication for trastuzumab therapy was early-stage and advanced/metastatic breast cancer. Age ≤ 18 or ≥ 75 years, structural heart disease, baseline EF ≤ 55%, severe hypertension, life expectancy ≤ 12 weeks, or abnormal renal or hepatic functions were exclusion criteria. Previous chemotherapy included regimens with ACs in the vast majority of cases. Patients received Trastuzumab intravenously on different schedules, according to different protocols. We collected: age, height, and weight, cardiovascular risk factors, cancer treatment details (in particular, previous ACs regimen, with dosage), and tumor characteristics.

All patients receiving chemotherapy at our Institution underwent EF measurement by echocardiography (modified biplane Simpson’s rule) at baseline (before therapy), every 3 months during the trastuzumab regimen, every 3 months during the first year after drug discontinuation, and every 6 months after that. In the case of cardiotoxicity, EF was measured monthly during the first 3 months of HF therapy. Nadir EF is the minimum value identified, while the final EF is the last available one.

All EF measurements were evaluated by two independent cardiologists, as in previous papers from our group (Cardinale et al., 2015). Any disagreement between the two readers (difference in EF>5 absolute points) was resolved by a joint evaluation of the echocardiographic findings.

Patients were assigned to standard care or to a cardioncological management strategy, according to the oncological referral center.

At our Institution, the targeted cardioncological follow-up consists of a thorough anamnestic overview (risk factors, cardiovascular history), an objective, electrocardiographic, and echocardiographic evaluation, biomarker evaluation (i.e., troponins, and BNP) and a precise and gradual introduction of cardioactive drugs (in particular angiotensin-converting-enzyme, A.C.E. inhibitors, and beta-blockers). Also, in patients managed with a targeted cardioncological approach, strict control of cardiovascular risk factors, in particular, arterial hypertension, was carried out. Also, in the same group, HF therapy was managed according to troponin and BNP values.

Exclusion criteria consisted of symptomatic HF during trastuzumab treatment or lack of substantial echocardiographic data.

### Outcomes

The primary endpoint was the occurrence of symptomatic HF (NYHA class II or higher). The diagnosis of HF was performed following the current clinical practice guidelines (Ponikowski et al., 2016).

The secondary endpoint was the end of trastuzumab therapy, after the diagnosis of trastuzumab-induced cardiotoxicity (TIC), following therapy rechallenge after cardioncological and oncological decision.

Follow-up data were retrieved using institutional medical records (outpatient clinic, oncologic and cardiologic follow-up) or by telephone contact (internal written informed consent signed at the baseline visit, as per institutional procedures).

### Statistical Analysis

General characteristics were classified using descriptive statistics.

Mean/median were used according to normal distribution. Comparisons were made using independent-samples t-test for continuous variables and Fisher’s exact tests for categorical variables. In order to test the independent determinants of EF variation (baseline–final), uni and multivariable regression analysis (Enter model; enter if p<0.05—remove if p >0.08 were performed.

Median disease-free survival was assessed with the Kaplan-Meier method (Log-Rank test).

p values < 0.05 were considered statistically significant.

All statistical analyses were performed with MedCalc 19.1.7 (Belgium, E.U.) and IBM SPSS Statistics for Windows, Version 24.0 (Armonk, NY: IBM Corp).

## Results

**Figure 1:**
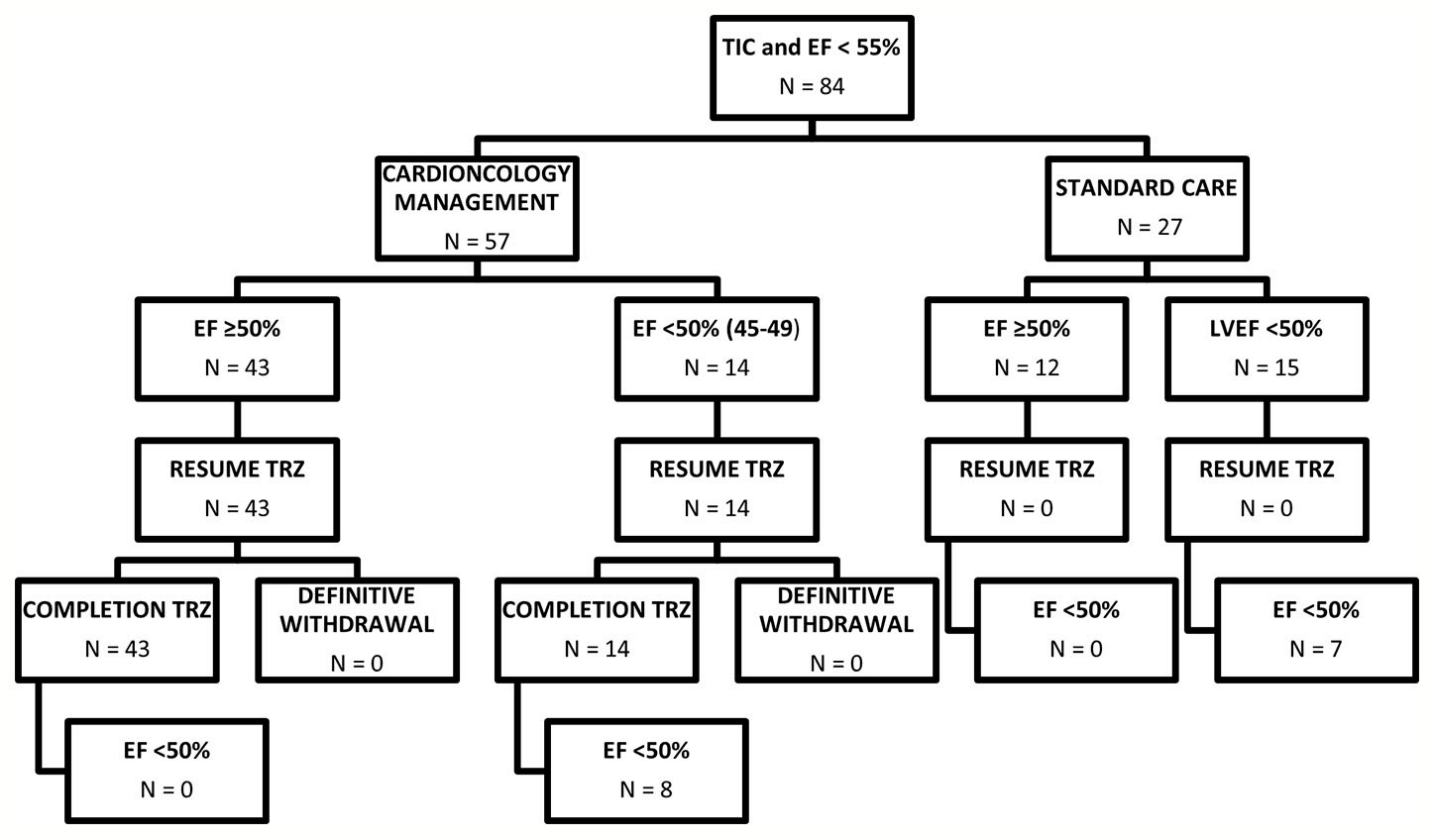
Diagram summarizing patients included in the study. EF, Ejection fraction; TIC; Trastuzumab induced cardiotoxicity; TRZ, Trastuzumab.

The final study population consisted of 84 female patients (age 51.7 years, 95% CI 49.5–53.8; **Figure 1**).

The characteristics of the population divided into standard care (27 patients) and cardioncological approach (57 patients) are summarized in **Table 1**.

**Table 1:** Population characteristics (n 84).

**Clinical Characteristics**	**Whole population (n 84)**	**Standard care (n 27)**	**Cardioncological approach (n 57)**	**p**
**Age, years**	51.7 (49.5–53.8)	52.5 (48.2–56.9)	51.2 (48.7–53.7)	0.5
**Height, cm**	165.3 (150.2–171.4)	163.3 (151.2–172.4)	166.2 (154.1–173.2)	0.7
**Weight, Kg**	54.2 (44.3–66.6)	55.5 (43.3–67.6)	54.1 (41.2–69.4)	0.5
**sCreatinine, mg/dL**	0.82 (0.71–0.97)	0.84 (0.72–0.99)	0.89 (0.78–1.02)	0.4
**Previous ACs**	76 (90.4%)	26 (96.3%)	50 (87.7%)	0.34
**Smoking History**	20 (23.8%)	6 (22.2%)	14 (24.5%)	0.9
**Arterial Hypertension**	23 (27.4%)	8 (29.6%)	15 (26.3%)	0.9
**Diabetes Mellitus**	1 (1.1%)	1 (3.7%)	0 (0%)	0.7
**Dyslipidemia**	14 (16.6%)	4 (14.8%)	10 (17.5%)	1
**Cardiovascular History**	3 (3.5%)	1 (3.7%)	2 (3.5%)	0.5
**Family history of Heart Disease**	15 (17.8%)	3 (11.1%)	12 (21%)	0.4
**Adriamycin, g/m^2^**	245.3 (235.3–255.3)	236 (216.2–255.7)	250 (238.1–261.8)	0.1
**Epirubicin, g/m^2^**	396.9 (330.4–463.4)	420 (314.6–525.3)	388.8 (301–476.3)	0.7
**Radiotherapy**	38 (45.2%)	11 (40.7%)	27 (47.3%)	0.56
**Surgery**	58 (69%)	19 (70.3%)	39 (68.4%)	0.85
**Adiuvant Setting**	33 (39.3%)	11 (40.7%)	22 (38.5%)	0.85
**Left Side Cancer**	48 (57.1%)	16 (59.2%)	32 (56.1%)	0.78
**Subcutaneous Trastuzumab**	63 (75%)	20 (74%)	43 (75.4%)	0.9
**Trastuzumab resumed**	57 (67.8%)	0 (0%)	57 (100%)	**<0.0001**
**ACEi-s +/- Beta-Blockers**	57 (67.8%)	12 (44.4%)	45 (79%)	**0.001**

ACEi-s, angiotensin-converting enzyme inhibitors.

Data are reported as n (%) or mean/median (95% Confidence intervals, CI). In bold: statistically significant.

The distribution of cardiovascular risk factors and cardiological history were overlapping between the two groups of patients i.e. the history of prior ACs exposure, disease setting (adjuvant/neo-adjuvant), radiotherapy exposure, and breast cancer site (all p ns) (**Table 1**).

No significant differences were observed, at baseline, in terms of anthropometric characteristics or renal function. In 73 cases, the diagnosis was early-stage whereas in 11 cases it was metastatic breast cancer.

Of the 73 patients 66 had been previously treated with anthracyclines (early-stage cancer) and 10 of the 11 advanced patients had received anthracyclines (metastatic disease). Both groups presented basal left ventricular systolic function values, evaluated in terms of EF %, preserved and overlapping (61.8, 95% CI 60–63.5% vs. 60.5, 95% CI 59.4–61.7%; p=0.5; **Table 2**).

**Table 2:** Echocardiographic parameters and Cardiac events (n 84).

**Clinical Characteristics**	**Whole population (n 84)**	**Standard care (n 27)**	**Cardioncological approach (n 57)**	**p**
**EF baseline, %**	60.9 (60–61.9)	61.8 (60–63.6)	60.5 (59.4–61.6)	0.2
**EF nadir, %**	44.5 (42.7–46.2)	40.6 (37.3–43.9)	46.3 (44.3–48.3)	0.002
**EF resume, %**	51 (50–52)	48.7 (46.7–50.6)	52.1 (51–53.3)	0.001
**EF final, %**	55.7 (54.4–57.1)	53.3 (50.6–56)	56.8 (55.4–58.2)	**0.01**
**ΔEF baseline–final, %**	5.2 (3.5–6.8)	8.6 (5.3–11.8)	3.7 (1.8–5.5)	**0.005**
**Time EF% Nadir to recovery**	19.7 (18–21.4)	20.8 (16.8–24.8)	19.2 (17.5–21)	**0.4**
**Cardiac Events**	13 (15)	7 (26)	6 (10)	**0.1**
**HF**	11 (13)	6 (22)	5 (9)	
**Pulmonary Edema**	2 (2.3)	1 (4)	1 (2)	

EF, ejection fraction.

Data are reported as n (%) or mean/median (95% Confidence intervals, CI). In bold: statistically significant.

We observed a decline of EF values during trastuzumab therapy (44.5, 95% CI 42.7–46.2%; p<0.0001).

After EF reduction (<50%), HF treatment—ACE-inhibitors and beta-blockers, possibly in combination—was promptly initiated, and increased to the maximally tolerated dose in all patients of the cardioncological approach group. All these patients (n=57) resumed and completed trastuzumab therapy (p<0.0001; n=47; 82% in the early-stage group; n=10; 91% in the metastatic group (*p*=0.01). No patients of the standard care group, even those who improved their cardiac function to a >50% EF value, resumed Trastuzumab. The decision not to resume oncological treatment with Trastuzumab was oncological 15 cases (55.5%), due to EF < 50% in five cases (18.5%) and in seven cases (26%) the patients’ personal choice.

The nadir of EF % observed during trastuzumab therapy was lower in the standard care group (40.6, 95% CI 37.3–43.9% vs. 46.3, 95% CI 44.3–48.3%; p=0.002).

The delay of treatment resumption after EF reduction was 45 days (min 21, max 94).

Patients in the standard care showed lower EF values at trastuzumab rechallenge (overall 51, 95% CI 50–52%; standard 48.7, 95% CI 46.7–50.6% vs. cardioncological follow-up 52.1, 95% CI 51–53.3%; p =0.001).

Median follow-up (months) was similar (59.5, 43.3–71.9 vs. 61.5, 46.9–76.1; p=0.1).

**Figure 2:**
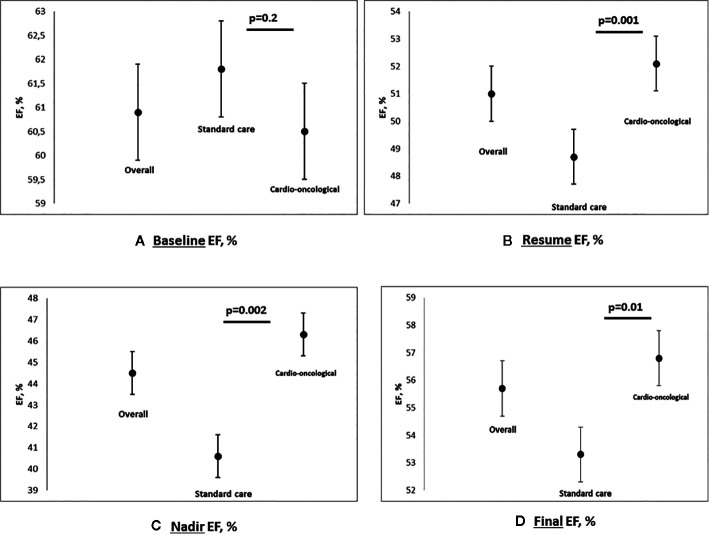
Multipanel figure: scatter plot for EF baseline **(A)**, nadir **(B)**, resume **(C)** and final **(D)**, % - Data are reported for overall population, standard care and cardioncological approach group (mean and SD, standard deviation; p values).

An overall reduction of EF was observed at the final evaluation (overall 55.7, 95% CI 54.4–57.1%, p<0.0001; standard 53.3, 95% CI 50.6–56% vs. cardioncological follow-up 56.8, 95% CI 55.4–58.2%; p=0.01; in **Figure 2** EF scatter plots—baseline, nadir, resume, and final- according to groupings).

At rechallenge, 49 (75.4%) in the cardioncological approach group vs. 12 (44.4%) in the standard care group, showed EF values >50% (p<0.0001).

Seven patients (25%) in the standard care group, vs. 8 (14%) in the cardioncological group, showed final EF values persistently below 50% (p=0.3) (**Figure 1**).

Mean time from nadir to final EF in patients who recovered was 19.7 months (95% CI 18–21.4), and no differences were reported according to management strategy (19.2, 95% CI 17.5–21 months vs. 20.8, 16.8–24.8 months; p=0.4). ΔEF baseline to final % was higher in standard care group (8.6, 95% CI 5.3–11.8 vs. 3.7, 1.8–5.5; p=0.005). At multiple regression analysis, accounting for cardioactive therapy (Beta -3.7; Std Err. 1.9; p=0.05) and cardioncological approach (Beta -4.8; Std Err. 1.9; p=0.005), the tailored approach was the only independent determinant of ΔEF baseline to final %(R^2^0.12; p=0.004).

We observed a difference regarding the total number of HF events. In the standard care group 7 (26%) vs. 6 (10%) in the cardioncology approach group but the difference is not statistically significant (p=0.1; **Table 2**).

**Figure 3:**
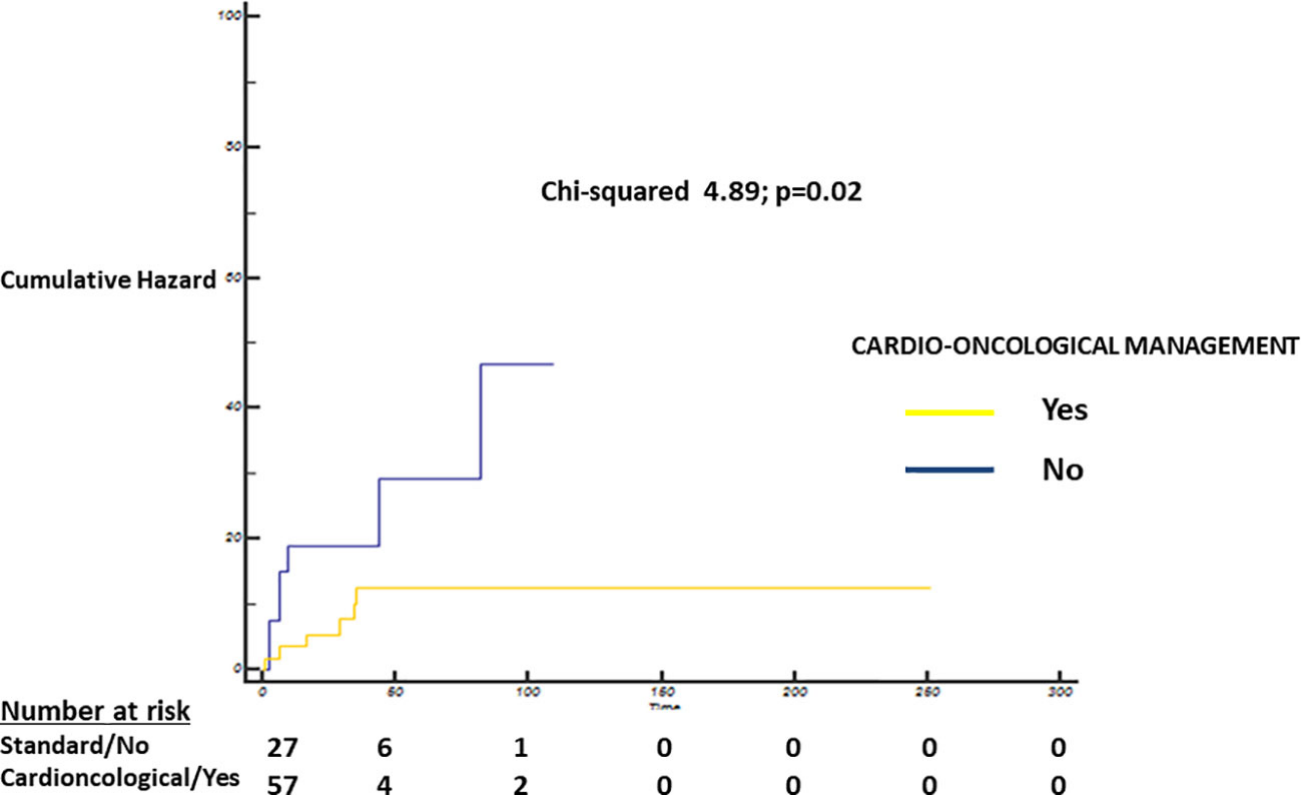
Cumulative Hazard—Log-Rank test showing a statistically significant difference between patients with standard care (blue) and intensive cardioncological (yellow) management strategy (standard care strategy 78.4, 95% CI 59–97 months vs. 98, 95% CI 89.7–106.2 months; Chi-squared 4.89; p=0.02).

Log-Rank test showed a statistically significant difference in terms of overall survival probability between patients with standard care and intensive cardioncological approach (standard care 78.4, 95% CI 59–97 months vs. 98, 95% CI 89.7–106.2 months; Chi-squared 4.89; p=0.02; **Figure 3**).

In patients managed with a dedicated cardioncological strategy, there was a significantly higher percentage of the introduction of cardio-active therapeutic regimens, including ACE inhibitors and beta-blockers (individually or in combination), without significant side effects (44.4% vs. 79%; p= 0.001).

Oncological overall free survival was higher in patients managed with the cardioncological strategy, who completed the trastuzumab regimen (p<0.0001).

## Discussion

In the present study, conducted on a population of 84 female patients with breast cancer suitable for trastuzumab therapy with evidence of asymptomatic cardiotoxicity, a dedicated cardioncological approach, with close clinical and imaging assesment (including prompt HF treatment) allowed individuals to resume and complete trastuzumab treatment, with fewer HF events and better overall survival.

Even if the baseline EF values were similar between the two groups, patients who did not undergo a cardioncological approach showed lower EF values at nadir, rechallenge, and at the final evaluation.

Our study confirms that trastuzumab therapy has an unfavorable impact in terms of ventricular function and cardiovascular events (Plana et al., 2014; Armenian et al., 2017) and it is essential to note that this patient population, also when managed with a dedicated clinical approach, remains potentially at risk of cardiac events, more than in healthy subjects, despite the reduction observed with a dedicated strategy.

To date, early and targeted treatment of asymptomatic left ventricular dysfunction are of primary importance to prevent further clinical events (Cardinale et al., 2010a).

In particular, evidence indicated that treatment with Trastuzumab seems to be associated with relevant alterations in cardio-pulmonary function (exercise; myocardial deformation imaging) that can promote the development of cardiac adverse events over time, suggesting the need for prompt initiation of HF therapies (Yu et al., 2020).

In fact, previous evidence from our group, although specific for ACs regimens, has demonstrated that early treatment with neurohormonal antagonist drugs, when left ventricular dysfunction was detected, is associated with a higher probability of recovery and a reduction in cardiovascular events (mainly in the first year after the detection of functional impairment) (Cardinale et al., 2010b).

Of particular importance, the targeted cardioncological approach has allowed not only the resumption but also the completion of trastuzumab therapy, contributing to a higher number of patients with normal or near-normal ejection fraction but also the reduction of events in the same population, with better oncological outcomes.

This confirms the findings of a recent paper by Hussain et al., conducted on a similar population (60 patients with evidence of TIC), which showed that continuation of Trastuzumab after an EF decline to < 50% is a promising approach, and a dedicated cardioncological service can substantially aid in this task (Hussain et al., 2019).

Further, the SAFE-HEaRt study is currently evaluating the safety of trastuzumab therapy in asymptomatic with reduced EF (Lynce et al., 2017).

Of course, patients with metastatic disease, despite evidence of reduced left ventricular function, more easily resumed and ended trastuzumab therapy.

In the cardioncological approach, as in our Institution, a gradual and progressive, but continuous introduction of cardiological therapies is practiced: this approach is proposed from the initial detection of left ventricular dysfunction, although mild and asymptomatic.

It is well known that this stage represents, even if early, an already advanced stage of damage induced by chemotherapy (later than one detected with a tailored biomarkers approach).

Although 14 patients failed to achieve an EF>50%, they still completed chemotherapy: this emphasizes that a close collaboration between cardiologist and oncologist can encourage more aggressive strategies in terms of chemotherapy, to the benefit of the patient. In particular, the oncologist is reassured by the cardiologist and may risk the continuation of potentially cardiotoxic therapies (Cardinale et al., 2018).

A current issue within the field in cardioncology is trastuzumab prosecution in patients with EF reduction due to therapy. The substantial survival benefit motivates this question in early-stage trastuzumab-containing regimens and metastatic patients (Piccart-Gebhart et al., 2005; Romond et al., 2005; Slamon et al., 2011; Balduzzi et al., 2014); moreover, clinical trials have failed to demonstrate the non-inferiority of short trastuzumab treatment durations (<12 months). These findings may thus support the continuation of this treatment strategy in early-stage patients even after EF reduction. In metastatic patients, Trastuzumab increases overall survival, with observations of sustained responses to therapy for as long as 9 years with no signs of disease progression (Cancello et al., 2008; Balduzzi et al., 2014). In particular, where the aim of the treatment is palliative, the reason for the continuity of the treatment might be more relevant.

Also, despite the continuation of Trastuzumab, the patients managed with the cardioncology approach presented higher EF values at the end of the treatment compared to standard management, such as fewer cardiovascular events, while allowing the continuation and conclusion of the chemotherapy program, with clear potential positive implications in terms of oncological outcome and cardiovascular events.

### Limitations

The limitations of the study are represented by its retrospective nature and in particular the low number of patients; however, the latter is comparable to recent reports from high volume centers.

Furthermore, we cannot extract information regarding ‘only trastuzumab therapy patients’, due to the high percentage of patients undergoing anthracycline therapy.

There is a lack of information on cardiac biomarkers such as troponin and natriuretic peptides: these have a significant potential to identify possible cardiotoxicity earlier. However, this information, particularly for patients not closely followed at our institute, was not recorded. As far as echocardiography is concerned, data on myocardial deformation indices (such as global longitudinal strain) are missing (mainly in the years 2010-2015), and the apparent limits of a left ventricular functional evaluation based on two-dimensional EF, even if validated longitudinally by a single operator, is also a limiting factor.

## Conclusions

A targeted cardioncological approach, in patients with evidence of cardiotoxicity during HER-2 inhibitor therapy, can favorably influence the oncological management of breast cancer patients, enabling the completion of active treatment regimens and reducing the adverse impact of chemotherapy on left ventricular function, with a reduced effect on HF events, and a higher rate of survival.

## Data Availability Statement

The raw data supporting the conclusions of this article will be made available by the authors, without undue reservation.

## Ethics Statement

The studies involving human participants were reviewed and approved by IEO - MILAN ITALY. The patients/participants provided their written informed consent to participate in this study.

## Author Contributions

DC, IF, NC, and CC contributed to the conception and design of the review and wrote the first draft. All authors contributed to the article and approved the submitted version.

## Conflict of Interest

The authors declare that the research was conducted in the absence of any commercial or financial relationships that could be construed as a potential conflict of interest.

## Abbreviations

ACE, Angiotensin-converting-enzyme; ACs, anthracyclines; EF, ejection fraction; HF, heart failure; HER-2, human epidermal growth factor receptor-2; TIC, trastuzumab-induced cardiotoxicity; TRZ, Trastuzumab.
